# Multi-omics analysis of an immune-based prognostic predictor in non-small cell lung cancer

**DOI:** 10.1186/s12885-021-09044-4

**Published:** 2021-12-10

**Authors:** Yang Zheng, Lili Tang, Ziling Liu

**Affiliations:** 1 Jilin University First Hospital, Changchun, Jilin People’s Republic of China; 2 Institute of Military Cognition and Brain Sciences, Academy of Military Medical Sciences, Academy of Military Sciences, Beijing, People’s Republic of China

**Keywords:** NSCLC, Immune subtype, Immune checkpoint inhibition, Multi-omics analysis

## Abstract

**Background:**

Inhibitors targeting immune checkpoints, such as PD-1/PD-L1 and CTLA-4, have prolonged survival in small groups of non-small cell lung cancer (NSCLC) patients, but biomarkers predictive of the response to the immune checkpoint inhibitors (ICIs) remain rare.

**Methods:**

The nonnegative matrix factorization (NMF) was performed for TCGA-NSCLC tumor samples based on the LM22 immune signature to construct subgroups. Characterization of NMF subgroups involved the single sample gene set variation analysis (ssGSVA), and mutation/copy number alteration and methylation analyses. Construction of RNA interaction network was based on the identification of differentially expressed RNAs (DERs). The prognostic predictor was constructed by a LASSO-Cox regression model. Four GEO datasets were used for the validation analysis.

**Results:**

Four immune based NMF subgroups among NSCLC patients were identified. Genetic and epigenetic analyses between subgroups revealed an important role of somatic copy number alterations in determining the immune checkpoint expression on specific immune cells. Seven hub genes were recognized in the regulatory network closely related to the immune phenotype, and a three-gene prognosis predictor was constructed.

**Conclusions:**

Our study established an immune-based prognosis predictor, which might have the potential to select subgroups benefiting from the ICI treatment, for NSCLC patients using publicly available databases.

**Supplementary Information:**

The online version contains supplementary material available at 10.1186/s12885-021-09044-4.

## Introduction

Lung cancer remains the largest cause of cancer deaths globally [1]. With non-small cell lung cancer (NSCLC) accounting for over 85% of all lung cancer cases, the classification of NSCLC based on histology, primarily lung adenocarcinoma (LUAD) and squamous cell carcinoma (LUSC), has resulted in substantial improvements in disease treatment and control [2, 3]. Meanwhile, recent progress in high-throughput sequencing technologies have revealed vastly different mutational and immune landscape between LUAD and LUSC, and in each subtype itself [4–7]. Consequently, lung cancer treatment is no longer confined to the use of cytotoxic medications but has been modified to include a more individualized approach. Particularly, drugs targeting specific driver mutations or immune checkpoints, if available, now have a major role in the treatment of selected patient subgroups with LUAD or LUSC [1, 8].

Important driver mutations have been detected in genes including *EGFR*, *ALK* and *ROS1*, with a higher mutation frequency in LUAD over LUSC [9]. Tyrosine kinase inhibitors (TKIs) against these mutations are available, and tumors harboring these mutations initially respond well to TKIs [10–12]. Nevertheless, this unprecedented benefit is only evident in only a small percentage of patients [13]. As all tumors eventually develop resistance through various mechanisms, the drug resistance has accelerated the development of X-line (second-line and later) TKIs, whereas further subtypes of tumors with the aforementioned genetic alterations are being identified, yielding the importance of screening for patients more beneficial to existing TKIs [2–4, 14–16].

Immune checkpoint inhibition (ICI), namely treatment with antibodies against immune checkpoints (such as CTLA-4, PD-1, PD-L1, Tim-3, TIGIT, and Lag-3), belongs to the category of immunotherapy that functions by regulating the immune system [17, 18]. ICI has shown remarkable early success in many malignancies, including NSCLC [19]. Many studies have reported on the mechanism and treatment of NSCLC with the help of immunotherapy, which has increasingly become a hot spot in the field, especially represented by PD-1/PD-L1 inhibition (Fig. S1A). Some targets for immunotherapy have not yet been sufficiently tested, but trials are underway. While the enthusiasm concerning the immune classification of NSCLC is still building up, durable responses from immunotherapy occur uncommonly. It is critical and challenging to find biomarkers that can help clinicians identify individuals who will benefit from immune checkpoint inhibitors (ICIs) [4, 16]. Early attempts to investigate the efficacy of ICIs linked the therapeutic response to PD-L1 expression by immunohistochemistry (IHC) in NSCLC tumor samples [20]. But later randomized trials have shown conflicting results while using this marker to guide therapy in NSCLC [21, 22]. Although there have been multiple reasons accounting for why its insufficiency to predict response [23, 24], IHC expression of PD-L1 is still the finest biomarker for these cases [19, 25]. Additionally, tumor mutational burden (TMB) is a possible biomarker for ICI treatment effectiveness [26]. But the assessment of TMB is not a common practice for NSCLC patients now. The identification for more accurate and feasible biomarkers that are predictive of prognosis and response to therapy is critical for the selection of patients, especially when multiple therapeutic choices are available [24].

Integrative analyses of genetic and epigenetic alterations enable a more comprehensive understanding of the immune composition in many cancer types, including NSCLC. The landmark project of the Cancer Genome Atlas (TCGA) provides researchers worldwide with a convenient and easy to access approach to a large number of cancer patients and related genomics data. Oncologists may now stratify cancer patients into multiple subgroups using next-generation sequencing technologies, which can help guide therapy decisions [18]. Even so, simply utilizing the expression profiles of different immune checkpoints to predict the prognosis of NSCLC is insufficient, represented by inconsistent prediction power of overall survival (OS) status (Fig. S1B). Considering the heterogeneity in the immune infiltration status based on our analysis of the composition of 22 immune cells among patients with NSCLC in the TCGA database (Fig. S1C), which might also affect the response of corresponding immunotherapy, we first stratified by the immune components between LUAD and LUSC patients in TCGA. By analyzing genetic alterations, signaling pathway changes, DNA methylation patterns as well as the expression of immune checkpoints in different immune subgroups and screening hub genes, we constructed a prognostic predictor in different NSCLC subgroups, with the goal of improving NSCLC risk assessment and potentially stratifying individuals who might benefit from the ICI treatment.

## Materials & methods

### Data collection

NSCLC patients’ clinical information (a total of 1014 patients, including 513 LUAD patients and 501 LUSC patients) was retrieved from TCGA (Table S1), and the expression data of mRNA, lncRNA and miRNA in each patient were obtained from https://portal.gdc.cancer.gov/. All data were Level-3 grade and could be openly obtained as a training dataset. Non-silent mutation data (SNP and INDEL) of NSCLC patients were downloaded from http://xena.ucsc.edu/. The copy number segments (after removing germline CNAs detected by SNP Array 6.0 platform in all NSCLC patients were obtained from http://www.firebrowse.org/. Methylation450k gene methylation levels in NSCLC patients were downloaded from https://gdc.xenahubs.net. Moreover, the RNA-seq expression data of GSE31852, GSE43580 and GSE120622, along with the GSE136961 dataset involving PD-1 immune checkpoint inhibitor treatment, were downloaded from Gene Expression Omnibus database (https://www.ncbi.nlm.nih.gov/geo/, Table S2) and used as validation datasets. The workflow of this study was summarized in Fig. S2.

### Construction of keyword network

In the PubMed database of NCBI, all publications concerning the immunotherapy of NSCLC literatures reported from 2010 to 2020 were retrieved. The records without DOI or PMC records were removed, and the DOI information of included literatures was extracted. The keywords were extracted from the abstract texts of all literatures by VOSviewer 1.6.6 software, and then clustered based on the co-occurrence frequency of keywords in a single line to construct the keyword network [27].

### Prognostic value of immune checkpoint expression in NSCLC

Kaplan Meier Plotter (http://kmplot.com/analysis/) is a public database of mRNA microarrays containing five types of cancer (breast, ovarian, lung, gastric and liver cancer), from which information on gene expression and disease prognosis can be obtained [28]. It was used to verify the value of the expression of six immune checkpoints in the judgment of OS probability in 1144 lung cancer patients. PD-L1 corresponding probe 227458_at, Tim-3 corresponding probe 235458_at, CTLA-4 corresponding probe 236341_at, PD-1 corresponding probe 207634_at, Lag-3 corresponding probe 206486_at, and TIGIT corresponding probe 240070_at were selected. Parameter settings were “Split patients by auto select best cutoff” and “Array quality control: excluding biased arrays”, while other parameters were defaulted.

### Immune component decomposition and construction of immune subgroups

Using CIBERSORT (https://cibersort.stanford.edu/) [29], the expression scores of 22 immune cell types (LM22 immune signature) per patient were determined using mRNA expression data from NSCLC tumor tissue samples from the TCGA database. Set the parameters to model = absolute, permutation = 1000, disable quantile normalization for RNA-Seq data as recommended, and others by default. According to the relative expression ratios of different immune cells, the cell expression heat map was plotted using pheatmap R package (v.1.0.12). Spearman correlation coefficients between immune checkpoints and immune cells were calculated using cor function in R package corrplot (v. 0.84), and dot blot was plotted using R package ggplot2 (v.3.3.2).

To identify robust clusters, the nonnegative matrix factorization (NMF) was performed. Unsupervised clustering by R package NMF (v.0.21.0) was performed for all TCGA-NSCLC tumors samples. To normalize estimated expression counts, DESeq2 (v.1.16.1) was employed, followed by a pseudo-count and log 2 transformation. Clustering of tumor samples was based on the LM22 signature genes. The optimal rank was determined using the default settings by 10 random runs. The final NMF clustering solution was obtained by 50 times run using the optimal rank. The prcomp package was used to perform principal component analysis (PCA). The first two principal components were selected to create the PCA diagram, and the sample points were colored according to the NMF clusters.

### Characteristics of tumor immune subgroups

Semi-supervised analysis was performed based on all LM22 immune characteristic genes. The reduceDimension function in the monocle package (v.2.18.0) was used, and manifold learning was performed based on the ‘Reversed graph embedding’ algorithm to construct the pseudotime trajectory of all immune characteristic genes. Then, the pseudotime value of each NSCLC sample was calculated, and the sample was projected into the climbing trajectory of MRS (marginal rate of substitution) estimation, that is, the slope of the non-difference curve [30].

The tumor purity and immune score of the samples were calculated by the estimateScore in the ESTIMATE R package (v.1.0.13) with default settings, where higher scores refer to greater immune components. StromalScore represented the stroma component score, while ImmuneScore the immune component score, and ESTIMATEScore the score of integrated stroma component score and immune component score. These were the general indicators reflecting the level of immune infiltration and immune degree. TumorPurity could reflect the proportion of tumor cells. The higher the tumor purity was, the lower the immune infiltration was. Comparison between the two groups was based on the stat_comparison_means function in the R package ggpubr (v.0.4.0), and the wilcoxon test was used to perform the statistical test of Mean Comparison *P*-values.

The survival of patients was analyzed using the default parameters of the survival package (v.3.2.7) and the survminer package (v.0.4.8). The ggsurvplot function generated the survival curve and the survfit function constructed the association between patient survival time and NMF subgroups. The t test was used to compare two groups, and one-way ANOVA was used to compare sample mean values across many groups.

### Single sample gene set variation analysis (ssGSVA)

The MSigDB database (https://www.gsea-msigdb.org/gsea/msigdb/index.jsp) was used to obtain the immune signature file, and the gsva function in R package GSVA (v.1.38.0) was used under parameters (method = ‘ssgsea’, kcdf = ‘Gaussian’, abs.ranking = TRUE). ssGSEA analysis was performed based on mRNA expression data [31, 32]. According to the normalized ssGSVA score matrix of each signaling pathway calculated by gsva, the heat map was drawn by pheatmap R package (v.1.0.12).

### Detection of driver genes

MutSigCV (v.1.41) could eliminate the interference of heterogeneity of mutations and discover cancer-related driving genes. Items with *P* < 0.05, q < 0.1 and *n* ≥ 5 were selected as cancer driver genes. The lollipopPlot2 function in R package maftools (v.2.6.0) was used to draw the lollipopPlot map of amino acid point mutation according to the mutation information of protein change in maf file. Moreover, the Spearman correlation coefficients between the driving gene and the immune checkpoint were obtained using the corrplot R package (v.0.84), and dot blot was plotted using R package ggplot2 (v.3.3.2).

### Copy number alteration (CNA) analysis

We used GISTIC 2.0 to analyze CNAs under parameters (−genegistic 1 -smallmem 1 -broad 1 -brlen 0.5 -conf 0.95 -armpeel 1 -savegene 1 -gcm extreme). Segment_Mean values higher than 0.2 was regarded as a gain, whereas less than − 0.2 was defined as a loss [33, 34]. The CoNVaQ web tool (https://convaq.compbio.sdu.dk/) was used to create a statistical model using Fisher’s exact test. IGV 2.4.19 (Integrative Genomics Viewer 2.4.19) was used to create CNA summary charts. The Spearman correlation coefficients between CNA-changed genes and immune checkpoint genes were calculated by using corrplot. The genes with |R| > 0.4 were selected, and the heat maps of CNA-changed genes and immune checkpoint genes in different subtypes were drawn by using R-pack pheatmap (v.1.0.10).

### Comparison of methylation levels

DNA methylation data were normalized with the R package wateRmelon (v.1.34.0) [35, 36]. And differential methylated probes were detected by the R package minfi (v.1.36.0). The Pearson Correlation Coefficient of gene expression associated to immune checkpoint methylation level was then calculated using the corrplot. The genes with |R| > 0.4 were selected, and the methylation levels of genes related to the methylation level of immune checkpoint in different subtypes were plotted using R package pheatmap (v.1.0.10).

### Differentially expressed RNAs (DERs) analysis

The limma package (v.3.46.0) was used to screen the differentially expressed lncRNAs (DElncRs), miRNAs (DEmiRs) and mRNAs (DEmRs) among subgroups, and items with *P* <0.05 and |logFC| >1 were regarded as DERs. To eliminate the heterogeneity between LUSC and LUAD, NMF1 VS NMF2A and NMF3 VS NMF2B were performed, and then the intersection of the DERs between the two was taken, and finally the DERs of immune subtypes were determined. Then GO/KEGG analysis was performed with the DAVID (v.6.8) database (https://david.ncifcrf.gov/) to annotate the biological significance of DERs. GO analysis of DERs enriched gene function, cell composition and biological process. KEGG analysis could analyze the important signaling pathways affected by DERs, and statistical significance was defined as a *P* value of less than 0.05.

### Construction of RNA interaction network

The miRNA targeted mRNAs were predicted using TargetScan (http://www.targetscan.org/vert 72/), miRDB (http://mirdb.org/), and miRTarBase (http://mirtarbase.cuhk.edu.cn/php/index.php). The regulatory relationship between miRNA and lncRNA (http://starbase.sysu.edu.cn/) was constructed by lncRInter (http://bioinfo.life.hust.edu.cn/lncRInter/) and LncRNA2Target (http://123.59.132.21/lncrna2target/). We then used online tools (http://bioinformatics.psb.ugent.be/webtools/Venn/) to draw Venn diagrams, according to mRNA-miRNA-lncRNA interaction relationship. The STRING database (https://www.string-db.org/) was used to query mRNA interaction relationship, and results were imported to cytoscape. The RNA regulatory network was constructed by calculating gene weight (degree) value.

### Prognosis model construction and survival analysis

The least absolute shrinkage and selection operator (LASSO) was used for the dimensionality reduction. The LASSO Cox regression algorithm is a variation of LASSO and was used to identify most related prognostic candidates. The LASSO regression model was used to screen hub gene genes related to prognosis, and to construct the survival risk prediction model. Using R package glmnet (v.4.0.2), the DERs with degree ≥80 were selected with “family = cox, s = 0. 01”. Then the COX model was constructed using the coxph function in the survival package (v.3.2.7), and DERs with high correlation with prognosis were further screened. To study patient survival, the default parameters of the survival package (v.3.2.7) and survminer program (v.0.4.8) were utilized. The survival curve was drawn by ggsurvplot function, and the forest map was drawn by ggforest function. The PrognoScan database (http://dna00.bio.kyutech.ac.jp/PrognoScan/index.html) was used to retrieve the prognostic effects of *CD19*, *GZMB* and *IFNG*. RiskScore = (− 0.1132305 * *CD19*) + (0.2073623 * *GZMB*) + (− 0.1267028 * *IFNG*). The critical risk value defined in this study was 1, with 1 as the grouping standard. If greater than 1, it was regarded to be in the high-risk group, and if less than 1, it was regarded to be in the low-risk group.

### Subcluster mapping

SubMap (v.3) was used to compare subclusters from two different cohorts on the GenePattern platform (http://genepattern.broadinstitute.org/) with default settings [37, 38]. Significant correspondences were determined with the cut-off value of *P* < 0.05 adjusted by Bonferroni.

### Statistical analysis

Using statistical software R (v.4.0.0) for statistical analysis and graphical visualization of all data. Unless otherwise stated, the significant level was set to 0.05. The t-test was used to compare measurement data with normal distribution between the two groups. To compare the mean values of samples across various groups, a one-way ANOVA was utilized. Count data used rank sum test. Benjamini-Hochberg analysis was used for correction after multiple tests. The specific statistical analysis could refer to the above sections.

## Results

### Molecular immune subtypes based on the LM22 signature genes in TCGA-NSCLC

**Fig. 1:**
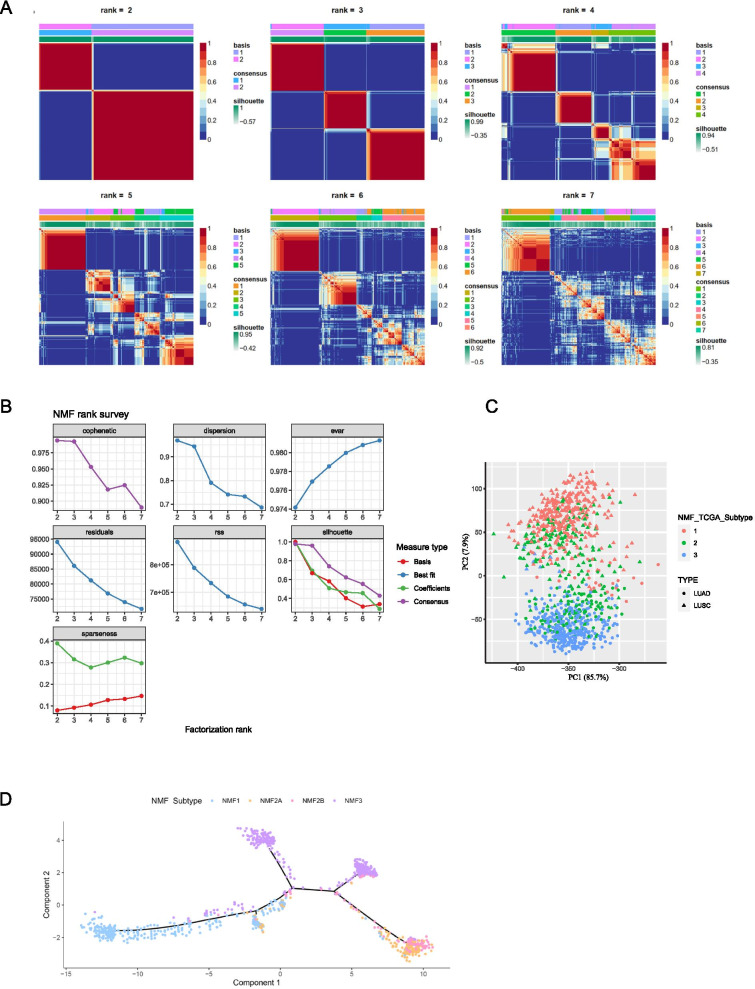
Construction of NSCLC immune subgroups by NMF classification. **A** NMF of the NSCLC cohort using the LM22 signature gene provided by CIBERSORT revealed better categorization when rank = 2 or 3; **B** Determination of k value using the NMF rank survey with multiple parameters; **C** Principal component analysis (PCA) using the first two PCs, PC1 and PC2, indicated that most NSCLC samples were PC1 < 0, which was then divided into two major groups by PC2. NMF subgroups and different pathological groups could be separated by PC2. Percentages in parentheses represented percent variance explained; **D** Monocle analysis constructed a pseudo timeline of immune characteristic genes, showing divergent immune composition of each NMF subgroup

1014 samples retrieved from TCGA-NSCLC, including LUAD (*n* = 513) and LUSC (*n* = 501), were used as a training cohort, which was analyzed by CIBERSORT (LM22) to assess the absolute amounts of distinct immune cell subtypes within individual samples. When rank = 2 or 3, meaning when NSCLC patients were separated into two or three groups, NMF results revealed improved categorization (Fig. 1A). Based on the NMF rank survey, we selected rank = 3 to divide these samples into three subgroups, named NMF1, NMF2 and NMF3 (Fig. 1B).

The PCA analysis showed that all samples were PC1 negative (Fig. 1C). Most NMF1 cases were PC2 positive, and most NMF3 were PC2 negative, whereas NMF2 was separated into PC2 positive and PC2 negative. These results indicated that the samples that underwent NMF grouping had significantly different spatial distribution. Interestingly, we found that PC2 could efficiently divide NSCLC cases into LUAD and LUSC (Fig. 1C), which was largely coincident with the NMF grouping. The NMF1 subgroup mainly consisted of LUSC patients 92.24% (333 / 361); while most LUAD patients 96.23% (357 / 371) fell to the NMF3 subgroup; in addition, the proportion of patients with either LUAD or LUSC in the NMF2 subgroup did not differ greatly: 45.39% (128 / 282) with LUAD and 54.61% (154 / 282) with LUSC. Therefore, we combined NMF grouping and the pathological types of the patients and subdivided the NMF2 sub-cluster into NMF2A (pathological type as LUSC) and NMF2B (pathological type as LUAD).

Since NMF typing was based on the LM22 immune signature, we examined the pseudotime axis of immune signatures in different subgroups using the Monocle analysis. NMF2A and NMF2B groups were at the developing end of the spectrum, indicating that their immune components were highly active and that there might be an active immune response; the NMF3 subgroups divided into two different groups, which might reflect the immune heterogeneity within the NMF3 group, but the intragroup differences were much smaller than the intergroup ones and were not distinguished; moreover, the NMF1 group exhibited the least active immune components (Fig. 1D). Together, these analyses identified four subgroups based on the NMF typing, indicating different immune status among NSCLC patients.

### Differences of immune cell distribution, signaling pathways, and clinical prognosis among NMF subgroups

**Fig. 2:**
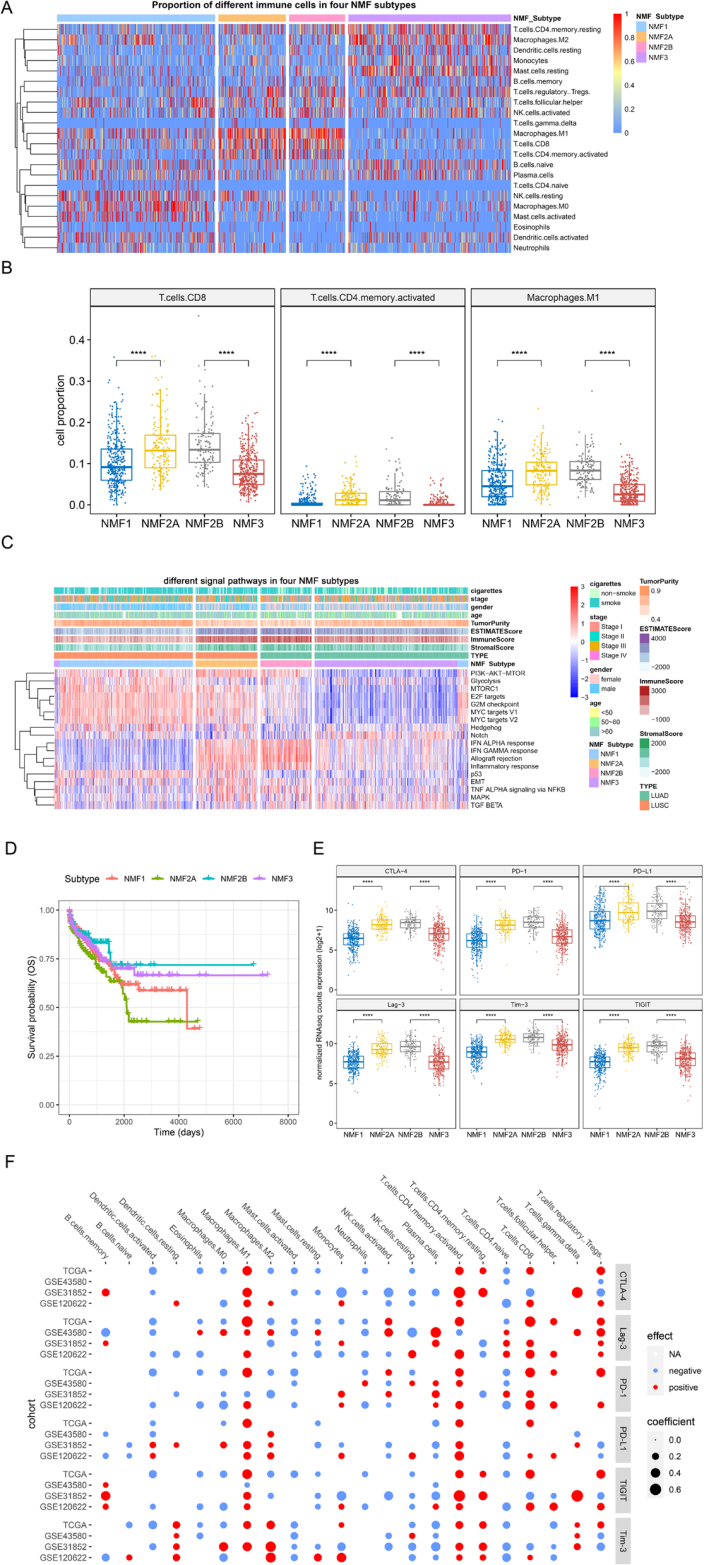
Immune cell composition, signaling pathway enrichment and expression of immune checkpoints among NMF subgroups. **A** Heat map of immune cell proportion revealed distinct composition among four NMF subgroups; **B** A higher proportion of CD8 T cells, activated CD4 memory T cells, and M1 macrophages was found in the NMF2 subgroup. Comparisons were made between NMF2A and NMF1, and between NMF2B and NMF3, separately; **C** The single sample gene set variation analysis (ssGSEA) showing enriched signaling pathways in four NMF subgroups; **D** Overall survival (OS) analysis indicated no significant difference in these immune groups based on the NMF classification; **E** The relative expression of six immune checkpoints was higher in the NMF2 subgroup. Comparisons were made between NMF2A and NMF1, and between NMF2B and NMF3, separately; **F** Significant associations between the expression of immune checkpoints and several immune cell types were identified in the TCGA database and several GEO datasets, where red dots indicated positive association and blue negative. The higher the coefficient, the stronger the association. ****P < 0.0001

Based on individual immune cell scores, we further analyzed the differences in immune cell composition of different NMF subgroups in the training cohort and found distinct immune cell composition among NMF1, NMF2, and NMF3 groups (Fig. 2A). Compared with the other two groups: the NMF2 group had a significantly higher proportion of CD8 T cells, activated CD4 memory T cells, and M1 macrophages (Fig. 2B); in the NMF1 group, resting NK cells, M0 macrophages, and activated mast cells constituted a significantly higher proportion; and the NMF3 group had higher proportions of resting memory CD4 T cells, M2 macrophages, resting DC cells, monocytes, and resting mast cells (Fig. 2B).

In addition, we calculated the tumor purity of patient samples as well as the immune score in different immune subgroups (Fig. S3). The NMF2 group exhibited the lowest tumor purity, as well as the greatest stromal score and immune infiltration, the situation of which was completely the opposite for the NMF1 group, with the NMF3 group in the middle.

To understand signaling pathway alterations in different immune subgroups, we performed ssGSVA on samples from the training cohort. By comparing NMF1 with NMF2A, or NMF3 with NMF2B, respectively, the enrichment score of *IFN*α response, *IFN*γ response, allograft rejection, inflammatory response was the highest in NMF2, suggesting the possibility of higher immune activities, and lowest in NMF1; while that of mTORC1, E2F target, G2M checkpoint, MYC targets V1, MYC targets V2, and p53 signaling was significantly higher in NMF1 + NMF2A compared with NMF2B + NMF3, highlighting the differences between the PC2 positive and the PC2 negative (Figs. 1C & 2C). These results indicated that different immune signaling pathways were enriched in these subgroups.

In terms of clinical outcomes, comparisons between NMF2A and NMF1 or between NMF2B and NMF3 revealed non-significant results (Fig. 2D), suggesting that the immune status was not responsible for the prognostic differences without the stratification by treatments targeting these immune changes.

### Differences in expression of six immune checkpoint genes related to immune phenotypes

**Fig. 3:**
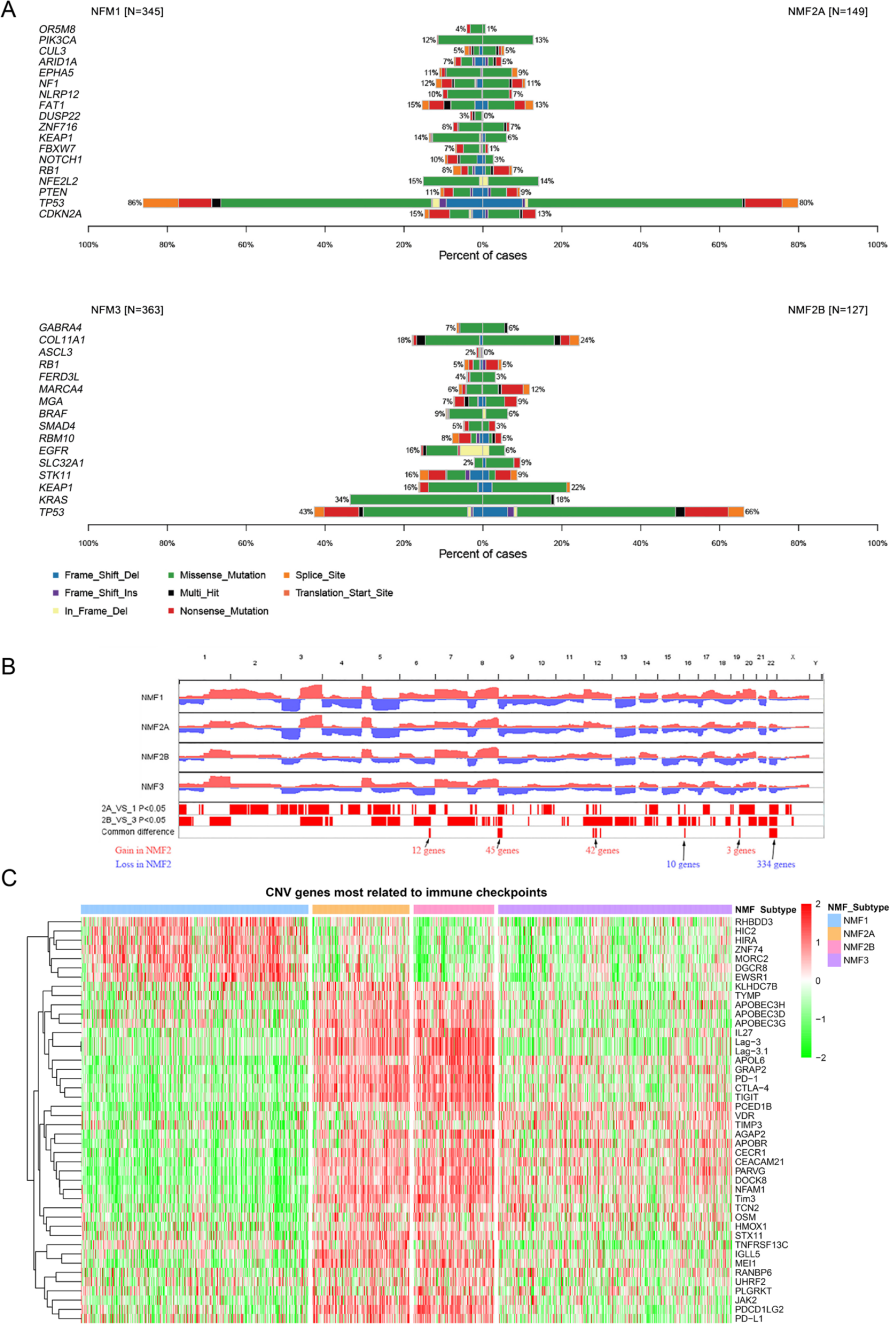
Mutation and copy number alteration (CNA) status varied among NMF subgroups. **A** Mutation frequency of driver genes in four NMF subgroups identified by MutSigCV; **B** CNA analysis indicated several hot spot regions with copy number gains and losses in the NMF2 subgroup. Comparisons were made between NMF2A and NMF1, and between NMF2B and NMF3, separately. The number of genes with CNA observed in these two comparisons was counted and shown; **C** Diverse expression patterns of genes with CNAs, showing correlation to immune checkpoints

**Fig. 4:**
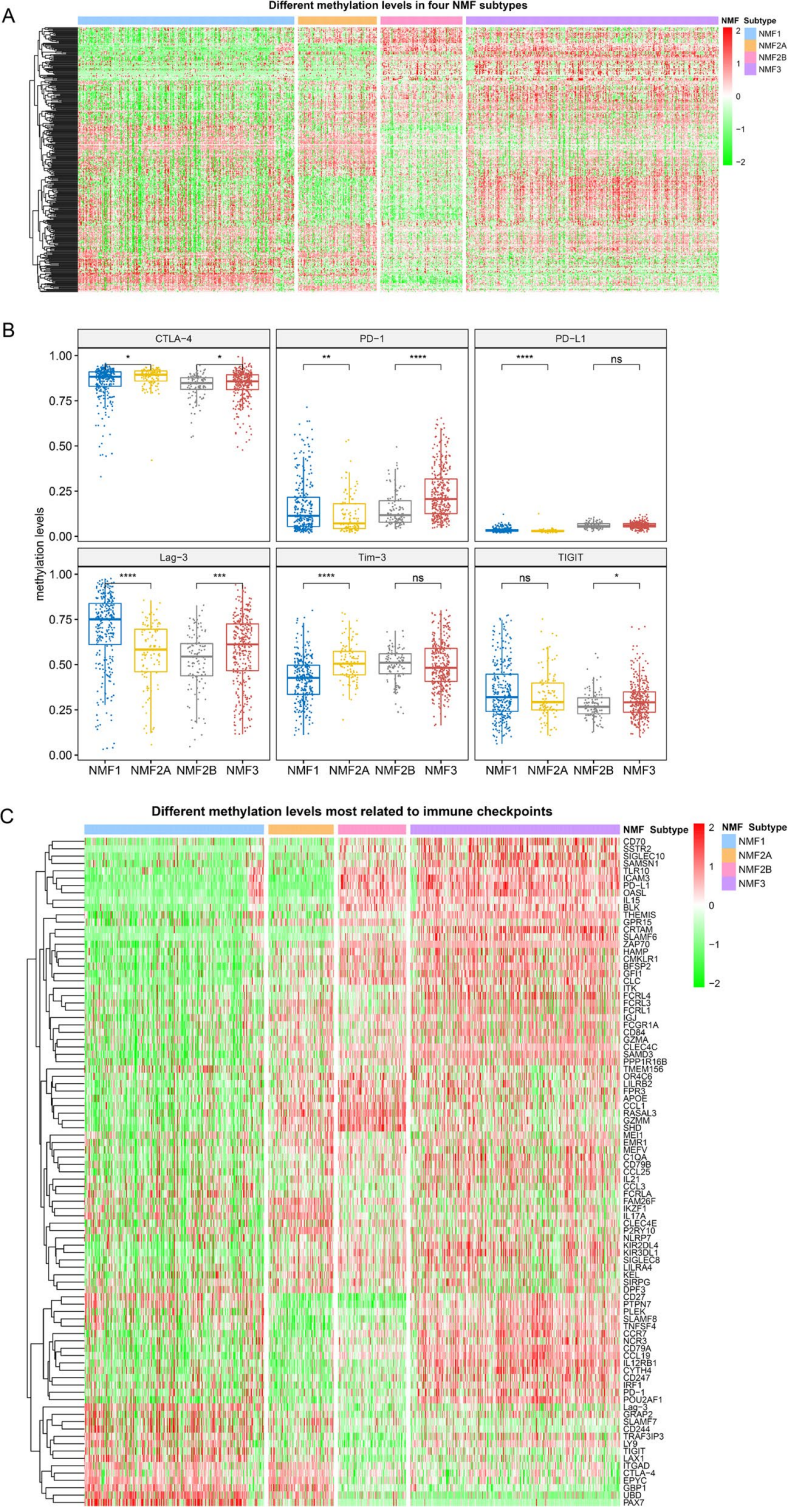
Methylation patterns among four NMF subgroups. **A** Heatmap of global methylation patterns in four NMF subgroups; **B** Inconsistent methylation levels identified in six immune checkpoints compared between NMF1 and NMF2A, and between NMF2B and NMF3; **C** Genes associated with immune checkpoints were found to be differentially methylated in different NMF subgroups. ns, non-significant; *P < 0.05; **P < 0.01; ***P < 0.001; ****P < 0.0001

The expression of immune checkpoints might be indicative of the clinical response of immunotherapies targeting immune checkpoints. Therefore, we next analyzed the expression profiles of the six important immune checkpoints (CTLA-4, PD-1, PD-L1, Tim-3, TIGIT and Lag-3) in different NMF subgroups. By comparing NMF1 with NMF2A, and NMF3 with NMF2B, the expression of the six immune checkpoints was significantly higher in NMF2A and NMF2B, respectively (Fig. 2E), which was not linked to the number of somatic mutations, but to CNAs of related genes, and might be partially associated with DNA methylation patterns (latter in Fig. S4B and Figs. 3C & 4C).

The link between immune checkpoint expression and immune cells in the training cohort was then investigated, as well as in three validation datasets. Statistically significant positive or negative associations between six immune checkpoints transcript levels and individual immune cell scores were computed by Spearman’s correlation and revealed three immune cell subtypes (CD8T cells, activated CD4 memory T cells, M1 macrophages) with a strong positive correlation in NMF2 (Fig. 2F). This is consistent with the elevated proportion of immune cells making up the immune repertoire of the NMF2 group (Fig. 2A), suggesting that these immune cells might have contributed significantly to the highly expressed immune checkpoints. Meanwhile, three subtypes (activated DC cells, M0 macrophages, activated mast cells) were found with an inverse correlation in NMF2 in most cohorts (Fig. 2F). And it was worth noting that M0 macrophages and activated mast cells were enriched in the NMF1 group (Fig. 2A), highlighting the different immune responses between patients in NMF1 and NMF2. These data suggested the possibility that expression of immune checkpoints might be important in distinguishing immune activities among NSCLC patients.

Together, the NMF2 sub-cluster could be termed as “immunoactive type” since it had higher CD8 T cells, activated CD4 memory T cells, and M1 macrophages; higher immune checkpoint expression; as well as *IFN*α response and *IFN*γ response, allograft rejection, and inflammatory response, while the NMF1 or the NMF3 termed as “immunoinactive type”.

### Differences in somatic mutations and CNAs related to the immune subgroups

To delineate the mutations of driver genes across immune subgroups, we used MutSigCV to examine driver genes and found that the most frequently mutated gene in the training cohort was *TP53*, but the mutation frequency of this gene was progressively decreasing from NMF1 to NMF3, at 86, 80, 66, and 43% (NMF1/NMF2A/NMF2B/NMF3), respectively (Fig. 3A). Comparison of *TP53* point mutations between patients in NMF1 and NMF3 also revealed variances in the location and number of mutations (Fig. S4A). In addition, we found that the mutation profiles between NMF1 + NMF2A and NMF2B + NMF3 were also obviously different. For example, in NMF1 + NMF2A, the common driver mutated genes included “*PTEN*”, “*NFE2L2*”, “*FAT1*”, while in NMF2B + NMF3, mutations in genes such as “*KRAS*” and “*EGFR*” were more common (Fig. 3A & S4A). These data suggested the difference of tumor driver mutations between PC2 positive and PC2 negative (Fig. 1C). However, the mutation patterns were similar between NMF1 and NMF2A, as well as between NMF2B and NMF3, even though there were some differences in the frequency of mutated genes (Fig. 3A). Between NMF2A and NMF2B, there were large differences in the frequency of driver genes. Considering the importance of expression of immune checkpoints for immunophenotyping, we calculated the correlation of expression between driver genes and immune checkpoints, and the results showed that the expression of several driver genes had both mutual exclusivity and co-occurrence; and the expression of six immune checkpoints exhibited positive correlation with each other; but between driver genes and immune checkpoints, except for a strong positive correlation between the expression of NLRP12 and Tim-3, few significant correlations were observed (Fig. S4B).

Recently, Davoli and colleagues provided strong evidence that somatic CNAs are associated with immune evasion, indicating a strong impact of genomic alterations on the tumor immune phenotype [39]. While NMF1 and NMF2A (or NMF2B and NNF3) shared similar patterns of CNAs, the overall quantity of CNAs in NMF2 was lower than that in NMF1 or NMF3. Analysis of genomic alterations revealed several hot spot regions with copy number gains (chromosomes 6, 9, 12 and 19) or deletions (chromosomes 16 and 22) as characteristic features of NMF2 as compared to NMF1/NMF3 in the training cohort (Fig. 3B). We compared the expression of all CNA-related genes to the expression of six immune checkpoints (Fig. 3C), and found significantly different expression patterns between the different subgroups, with the expression of genes such as *RHBDD3*, *HIC2*, *HIRAZNF74* being highest in the NMF1 group; with the expression of genes such as *IGLL5*, *MEI1*, *JAK2* was the highest in the NMF2 group, as well as that of six immune checkpoints; while in the NMF3 group, the expressions of all the above genes were lower. These data suggested that different expression of these six immune checkpoints in different NMF subgroups might be associated with genes with CNAs.

### Differences in DNA methylation related to the immune phenotype

To find out whether DNA methylation affects the development and maintenance of the NMF immune phenotypes, we analyzed global methylation data which were available for the training cohort. Methylation patterns varied among NMF subgroups (Fig. 4A), but DNA methylation at six immune checkpoints showed incompletely consistent alterations across these groups. For example, the lowest levels of methylation of *PD-1* and *LAG-3* were found in the NMF2 group compared to NMF1 and NMF3; *CTLA-4* showed higher methylation level in the NMF2A group compared to NMF1, but not in the NMF2B group compared with NMF3 (Fig. 4B). We plotted the methylation level heatmap of the genes associated with the methylation levels of these six immune checkpoints, and found that a subset of genes showed methylation patterns related to immunophenotyping, such as *CD27*, *PTPN7*, *PLEK*, *SLAMF8*, which had significantly lower methylation levels in the NMF2 group than in the NMF1 and NMF3 groups; whereas more genes showed methylation patterns related to the patient’s pathological type, such as *TLR10*, *ICAM-3*, *PD-L1*, which were considerably less methylated in the NMF1 + NMF2A group than in the NMF2B + NMF3 group (Fig. 4C).

### Identification of hub genes among NMF subgroups

**Fig. 5:**
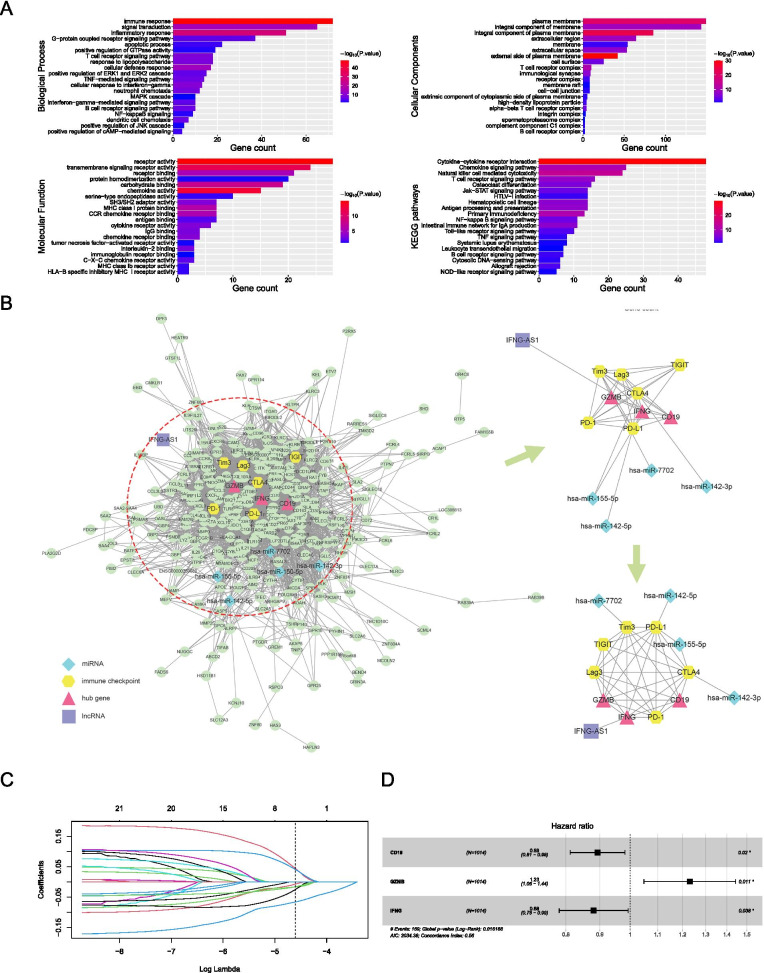
Identification of hub genes in NMF subgroups. A Taking the intersection of differentially expressed mRNAs (DEmRs) between NMF2A and NMF1, and between NMF2B and NMF3. GO/KEGG analyses of common DEmRs were shown; **B** The mRNA-miRNA-lncRNA network was constructed using the STRING database, and a sub-cluster was identified and rearranged; **C** Screening of potential hub genes using the LASSO regression model; **D** Forest plotting of three hub genes with significant hazard ratios. *P < 0.05

Even though the majority of patients derive clinical benefit from the ICI therapy, only a minority of them would experience durable/long-term responses, which makes the screening of hub genes that are predictive of response to ICI vital. By comparing NMF1 with NMF2A, or NMF2B with NMF3, we identified DEmRs, DEmiRs, and DElncRs in both comparisons. In total, 478 DERs were identified, including 346 mRNAs, 121 lncRNAs and 5 miRNAs with a significantly higher expression and 6 mRNAs with a lower expression in NMF2 (Fig. S5A-C**)**. Gene Ontology (GO) enrichment analyses confirmed that 346 upregulated mRNAs in NMF2 were related to immune response, as evidenced by enrichment in “innate/adapative immune response”, “signal transduction”, “inflammatory response”, “cytokine-cytokine receptor interaction”, “chemokine signaling pathway”, etc. (Fig. 5A).

We next intended to establish a mRNA-miRNA-lncRNA network based on the DERs in NMF2. We cross-referenced the DEmRs and the DElncRs identified here and the targeted mRNAs or lncRNAs of five DEmiRs predicted from different databases (Fig. S5D). Together with the mRNA interactions, we selected mRNAs and lncRNAs that were identified before and in at least one other database, and miRNAs to construct the mRNA-miRNA-lncRNA network, which would summarize underlying molecular traits of distinct tumor immune phenotypes (Fig. 5B, left). Using the LASSO regression model, we screened out seven DERs with the core node of action at a degree ≥80, *CTLA-4*, *CD19*, *GZMB*, *CD69*, *PRF1*, *IFNG*, and *PD-L1* (Fig. 5C). By the forest plot analysis of these seven genes, a lower hazard ratio was found for patients with higher expression of CD19 or IFNG (Fig. 5D), while that of GZMB or PRF1 corresponded to a higher hazard ratio (Fig. 5D & S6). These seven genes were put through a multivariate Cox regression analysis, and a three-gene Cox prognostic model was constructed, CD19-GZMB-IFNG, and these three genes were defined as hub genes in this study (Fig. 5D). We extracted the interactions of these three hub genes with six immune checkpoints from the network and constructed a sub-cluster and found that the three hub genes had direct associations with all immune checkpoints except that TIGIT was not directly linked to CD19 or IFNG (Fig. 5B, right).

A total of eight cohorts containing OS information of NSCLC patients (including LUAD and LUSC) were selected from the PrognoScan database (Table S3), and it was found that not any single one from three hub genes had significant association with patients’ OS.

**Fig. 6:**
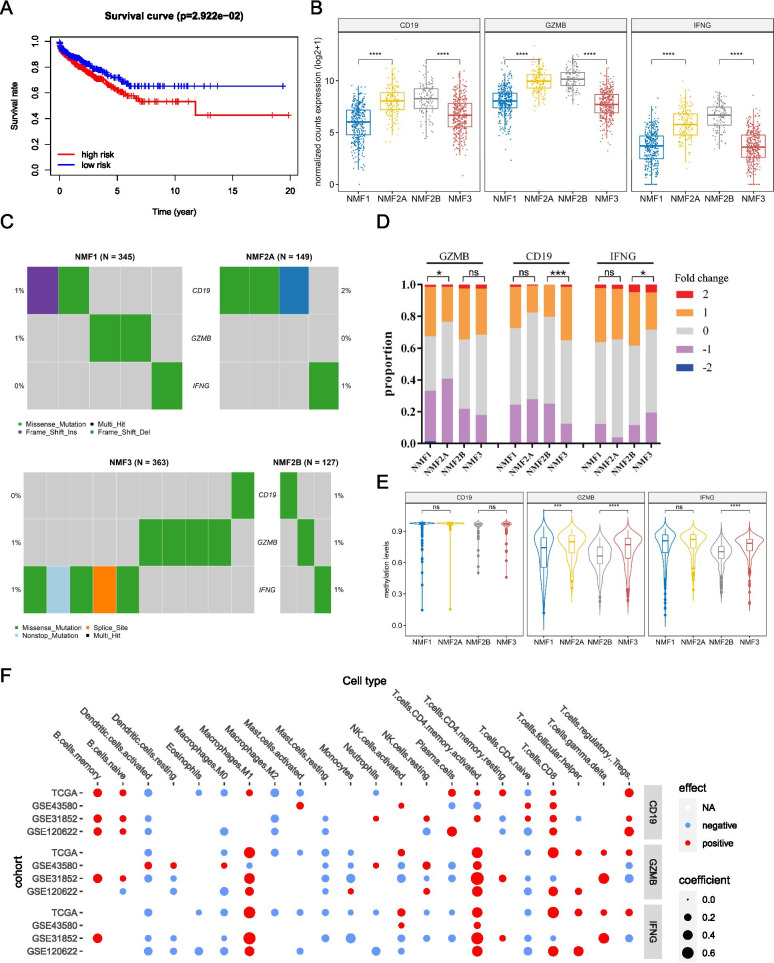
Validation of the three-gene prognostic predictor. **A** Survival analysis indicated a worse prognosis for the high-risk group, compared with the low-risk group defined by the risk score; **B** Higher expression of hub genes was observed in NMF2 compared with NMF1 and NMF3; **C** Non-significant mutation patterns of hub genes among NMF groups; **D** CNAs of hub genes in different NMF groups; **E** Inconsistent DNA methylation patterns of hub genes were identified; **F** All three hub genes were positively associated with M1 macrophages, T cells CD4 memory activated and CD8 T cells in the TCGA-NSCLC dataset and the three validation datasets. ns, non-significant; *P < 0.05; ***P < 0.001; ****P < 0.0001

We split the patients into high-risk and low-risk groups and compared the prognosis of the two groups using the aforesaid Cox model to produce a risk score for each NSCLC case in TCGA. We discovered that the model was capable of effectively partitioning and predicting patient survival: Patients in the high-risk group (greater CD19 and IFNG expression and lower GZMB expression) had a reduced survival time, while patients in the low-risk group (lower CD19 and IFNG expression and higher GZMB expression) had a longer survival time (Fig. 6A). We examined the expression of CD19, IFNG, and GZMB in different subgroups and found that the NMF2 group exhibited higher expression of all three genes than the other two groups did. (Fig. 6B).

We next examined these three hub genes in different subgroups for gene mutations, CNAs, and methylation level. The three hub genes had low mutation frequencies in different sub-groups, and none of them showed significant differences among groups (Fig. 6C), but the mutation sites were not the same in the patients in whom the mutations occurred (Fig. S7A). In addition, the expression of hub genes was not substantially linked with driver gene expression (Fig. S7B). We also found more copy number deletions of *GZMB* in NMF2A than in NMF1; and compared with that in NMF3, there were more *CD19* copy number deletions and more *IFNG* copy number gains (Fig. 6D). While the methylation level of *GZMB* in NMF2A was much greater than in NMF1, in NMF2B it was much lower than that in NMF3, as was *IFNG* (Fig. 6E).

We assessed the correlation of these three hub genes with immune cell constitution. All three hub genes were positively associated with M1 macrophages, T cells CD4 memory activated and CD8 T cells; might also positively correlate with B cell memory, T cells gamma delta; and negatively correlated with M2 type macrophages and mast cell resting (Fig. 6F).

### Validation of the three-gene prognostic signature

**Fig. 7:**
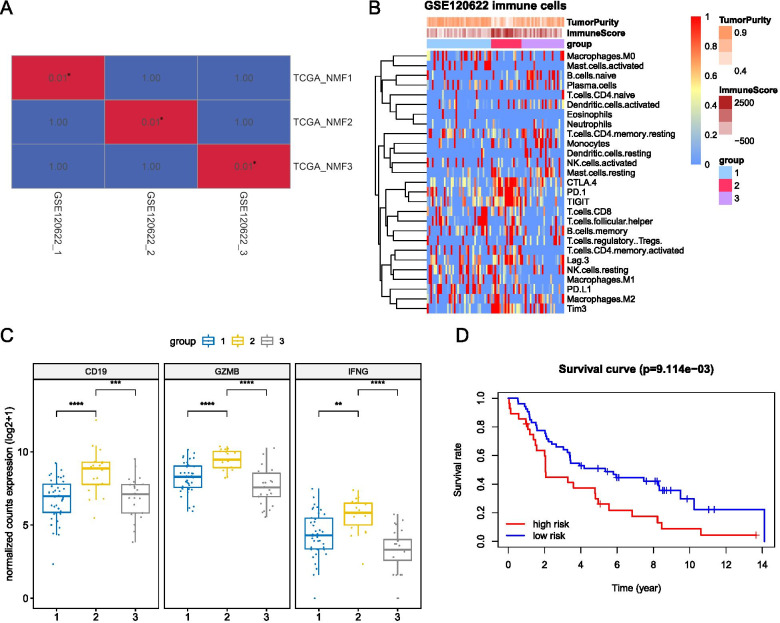
Validation of NMF classification and hub genes using the GSE120622 dataset. **A** Correspondence of NMF classification between GSE120622 and the training cohort by subcluster mapping; **B** Immune cell composition of the NMF subgroups identified in the validation dataset; **C** The relative expression of hub genes in three NMF groups in GSE120622; **D** Survival analysis using the prognostic predictor constructed earlier in GSE120622. ns, non-significant; *P < 0.05; **P < 0.01; ***P < 0.001; ****P < 0.0001

To confirm the findings in the training cohort, we applied the same NMF decomposition in the GSE120622 dataset, which yielded three immune subtypes: group1, group2, and group3, corresponding to NMF1, NMF2, and NMF3, respectively (Fig. 7A). Group2 had the highest immune scores and the lowest tumor purity scores; higher proportion of T cells CD4 memory activated and M1 macrophages (Fig. 7B); and higher expression of six immune checkpoints and three hub genes (Fig. 7B, C). After the Cox model divided the patients into high-risk and low-risk groups, the high-risk group showed a decreased survival probability (Fig. 7D).

To address the question, whether these three hub genes could serve as predictive biomarkers for ICI response, the GSE136961 dataset, which provided expression data for an immune gene panel for NSCLC patients treated with anti-PD-1 antibodies, was studied. Patients were separated into two groups based on their responses: (1) those who had a durable clinical benefit (DCB), defined as a partial or complete response to anti-PD-1 antibody by Response Evaluation Criteria in Solid Tumor (RECIST) v1.1 lasting > 24 weeks or stable disease lasting > 24 weeks, and (2) those who had a non-durable clinical benefit (NDB), demonstrating progression of disease or stable disease lasting ≤24 weeks [40]. Among the patients with DCB, they tended to express relatively higher expression of CD19, GZMB and IFNG (Fig. S8).

Together, this immune based three-gene signature was validated to be predictive for the prognosis of NSCLC patients and was associated with potential response to the ICI therapy. The comprehensive application of the Cox model in this study might be of great clinical importance for the risk management in NSCLC.

## Discussion

Risk assessment of prognosis and identifying patients who may benefit from current or potential ICI therapy is crucial [41]. Here, we constructed an immune-based stratification model for NSCLC patients using the NMF approach. Evaluation of the expression of six important immune checkpoints, as well as the evaluation of genomic alterations, signaling pathway enrichment and DNA methylation patterns, indicated specific subtypes with varied immunological background. Further screening identified *CD19, GZMB* and *IFNG* as hub genes of a complex RNA regulatory network associated with the immune status difference. This three-gene prognostic predictor improved the risk assessment of NSCLC patients and might be associated with the selection of patients who were more likely to benefit from the ICI therapy.

From pathological and mutational classification to immunological subtypes, in-depth tumor characterization enabled us to delineate the tumor entities and carry out corresponding precise treatment more accurately [42–45]. In this study, we combined the pathological status with the immune components to stratify NSCLC patients into the “immunoactive type” or the “immunoinactive type”, based on the immune cell makeup, immune checkpoint expression, and signaling pathway enrichment. Interestingly, we observed a small percentage of LUAD patients in NMF1 and that of LUSC in NMF3 (Fig. 2C). This small subset of cases with no good correspondence was made as a compromise to distinguish between groupings of different immune status and the pathological classification, and it also suggested that there might be patients with LUSC or LUAD whose immune status was similar to those with the opposite pathological status, which might be of clinical significance that the clinical advice for specific “immunoinactive” patients might rely solely on the immune status, regardless of the pathological phenotype.

Evaluation of immune cell composition in these subgroups revealed high percentage of CD8 T cells, activated CD4 memory T cells, and M1 macrophages in NMF2, all of which had important implications in regulating the immune response and the expression of immune checkpoints [46–50]. But the overall prognostic value of immune cell infiltration depends on many other factors, such as smoking status [51]. The higher proportion of these immune cells was not sufficient to maintain the immune response against tumor cells, since their functions might be inhibited or dysregulated by multiple mechanisms. And a less dysfunctional population of these cells might be crucial in initiating durable response to ICI [52]. The high expression of six immune checkpoints on these immune cells in NMF2 might serve as a potential immune escape mechanism by the tumor cells, therefore hindering the effectiveness of NMF stratification alone in determining the prognosis of NSCLC among different immune subtypes. The enrichment of *IFN*α response, *IFN*γ response, allograft rejection, inflammatory response pathways in NMF2 was also evidence of potential immune response and might be associated with the efficacy of the ICI therapy, posing a selective advantage over other groups with little expression of these immune checkpoints when considering the ICI therapy.

We further performed an integrative analysis of multi-omics data to highlight associations between tumor immune classifications and the genetic/epigenetic alterations. Analysis of somatic mutations among subgroups unraveled obvious differences between PC2 positive and PC2 negative, instead of between the “immunoactive type” and the “immunoinactive type”. These results suggested that somatic mutations might have limited contribution to the expression of immune checkpoints in different immune subtypes, and that these differences mainly stemmed from pathologic subtypes rather than immune status. *TP53* was the most frequently mutated gene throughout all immune subtypes, though at different frequencies. Even though not validated in our study, a previous report indicated that *TP53* mutation in LUAD could be used as a predictive factor for anti–PD-1/PD-L1 immunotherapy [42]. The role of specific point mutations in determining the response from the ICI therapy should be further evaluated.

Somatic CNAs are closely associated with tumor immune phenotype. Analysis of CNAs indicated most genes on chromosome 22 with copy number deletions in NMF2, compared with NMF1 and NMF3. While little is known about its implications in the prediction of ICI response, previous reports found an association between chromosome 22 loss and progression of NSCLC [53, 54]. Multiple CNA-containing genes have been shown to be tightly linked to immune checkpoint expression, such as the connection between *JAK2* and *PD-L1*. In addition, prior research has found a link between PD-L1 protein expression and amplification of the *PD-L1* and *JAK2* genes in NSCLC via the JAK-STAT signaling pathway [55–57]. Future investigation in CNAs of specific genes might prove useful in the prediction of ICI response.

In addition to genetic alterations, DNA methylation of genes, such as tumor suppressors, occurring during carcinogenesis and tumor development affects the immunogenicity and tumor response [58, 59]. In this study, we observed inconsistent methylation patterns in six immune checkpoints corresponding to their expression profile, and many genes with DNA methylation changes were associated with pathological status, instead of immune subtype. These data prompted us that although it might affect the expression of certain genes across different subgroups, the methylation patterns of immune checkpoints or related genes did not dominate the determination of NMF subgroups, and that targeting specific immune related genes or gene sets and examining their corresponding methylation levels might allow a more comprehensive elucidation of their expression differences across immune subtypes.

To efficiently extract genes that were stratified for prognostic risk in different immune subtypes, we analyzed DERs, including DEmRs, DElncRs, and DEmiRs. And by constructing regulatory networks associated with immune checkpoints, we identified seven hub genes, some of which are well-known regulators of immune surveillance [60–62]. By constructing the Cox model, we finally identified a three-gene signature for prognosis and risk assessment, namely *CD19*, *GZMB* and *IFNG*. High risk patients (greater expression of CD19 and IFNG, and low expression of GZMB) indicated a worse prognosis and might be more suitable for the use of ICIs. However, we also noticed that CD19, GZMB, and IFNG expression levels were considerably greater in the immunoactive NMF2 group compared with other groups, which might partially explain why the survival status of the NMF2, and other patient groups did not differ significantly. CD19 is presently thought to be the best accessible target for CAR-T cell treatment in blood cancer [63], whereas autophagic degradation of GZMB represents a novel method for hypoxic tumor cells to avoid natural killer cell-mediated lysis, and IFNG is important in maintaining immune homeostasis [64, 65]. The increased expression levels of GZMB and CD19 did not coincide with CNAs or altered methylation levels, suggesting the existence of other genomic or epigenetic effects; and despite the fact that higher IFNG expression was linked to CNAs or altered methylation levels, it certainly could not be excluded that there might be other factors affecting its expression. The mechanistic study concerning the association between CD19-GZMB-IFNG and ICI response remains to be further investigated. Nevertheless, these data raised the possibility that for patients with NSCLC, those with higher expression of hub genes might be “immunoactive”, have higher expression of immune checkpoints, and a higher CD4 memory activated and CD8 T cell repertoire, and they might be more sensitive to ICIs. Thereafter, it was possible that the risk degree and prognosis could be evaluated by the three-gene signature constructed here.

Similar to other bioinformatics approaches applying an integrative analysis of multi-omics data derived from bulk tumor tissue, this study shares some limitations. First, the limitation of study subjects. We mainly collected data from TCGA database as training data set, and a few GEO datasets for validation. The selection of the sources of these data introduced sampling bias, for which we lacked enough real-world data to testify our conjecture and to judge the validity of our three-gene signature. And the small sample size of the GSE136961 limited our interpretation of the utility of the prognostic predictor in assessing the risk and benefit of NSCLC receiving the ICI therapy. Second, while genetic and epigenetic alterations were evaluated more macroscopically for alterations related to immune checkpoint expression, there was no discussion of how individual genes impact the expression of immune checkpoints. Third, the lack of combination of the currently existing promising biomarkers indicative of response to the ICI therapy, such as the tumor mutational burden (TMB) [66], and the prognostic model identified in this study to reduce the potential bias of each one [23, 24]. Fourth, not every NSCLC patient is available to provide sufficient samples for sequencing, limiting the representativeness of currently sequenced tissues [67]. More attention should be paid in the above aspects to minimize the impact these limitations have on the findings.

In conclusion, our study provides an approach to construct a predictor using multi-omics data to evaluate the risk and the prognosis of NSCLC patients, which may act as possible indicators for identifying individuals who would benefit from ICI treatment.

## Supplementary Information

**Additional file 1.**

**Additional file 2.**

**Additional file 3.**

**Additional file 4.**

**Additional file 5.**

**Additional file 6.**

**Additional file 7.**

**Additional file 8.**

**Additional file 9.**

**Additional file 10.**

**Additional file 11.**

## Data Availability

The datasets generated and/or analysed during the current study are available as described in the Methods section.

## Abbreviations

NSCLC: Non-small cell lung cancer
LUAD: Lung adenocarcinoma
LUSC: Squamous cell carcinoma
TKIs: Tyrosine kinase inhibitors
ICI: Immune checkpoint inhibition
ICIs: Immune checkpoint inhibitors
IHC: Immunohistochemistry
TMB: Tumor mutational burden
TCGA: The Cancer Genome Atlas
OS: Overall survival
NMF: Nonnegative matrix factorization
PCA: Principal component analysis
MRS: Marginal rate of substitution
ssGSVA: Single sample gene set variation analysis
CNA: Copy number alteration
DERs: Differentially expressed RNAs
DEmRs: Differentially expressed mRNAs
DElncRs: Differentially expressed lncRNAs
DEmiRs: Differentially expressed miRNAs
LASSO: Least absolute shrinkage and selection operator
