# Supplementary figures and images for: Multi-omics analysis of an immune-based prognostic predictor in non-small cell lung cancer
Source: BMC Cancer. 2021 Dec 10;21:1322. doi: 10.1186/s12885-021-09044-4 (PMC8662860; doi:10.1186/s12885-021-09044-4)

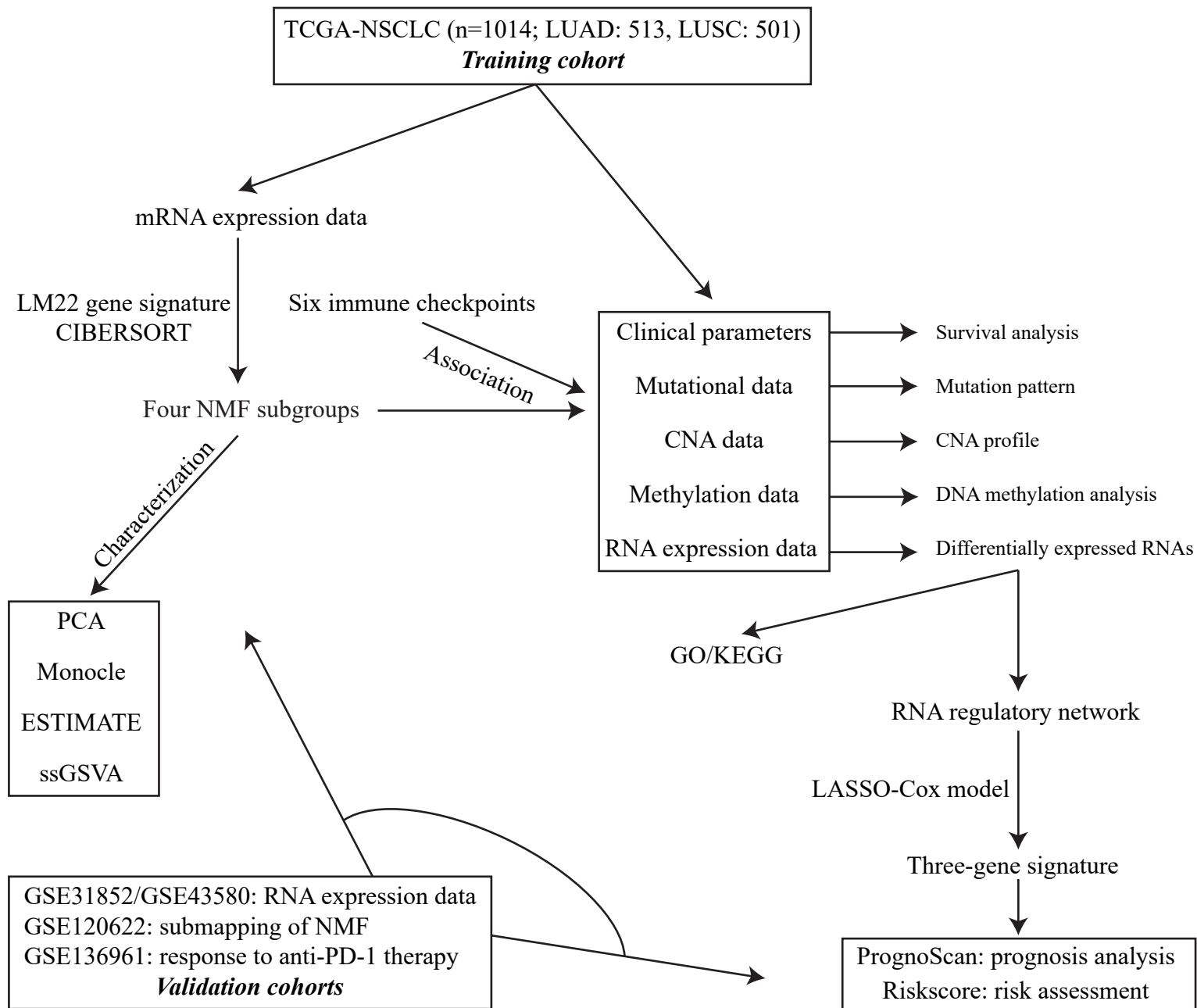

**Figure S2. The workflow of this study.**

Supplement: Supplementary file 4 — Additional file 4. [file 12885_2021_9044_MOESM4_ESM.pdf]

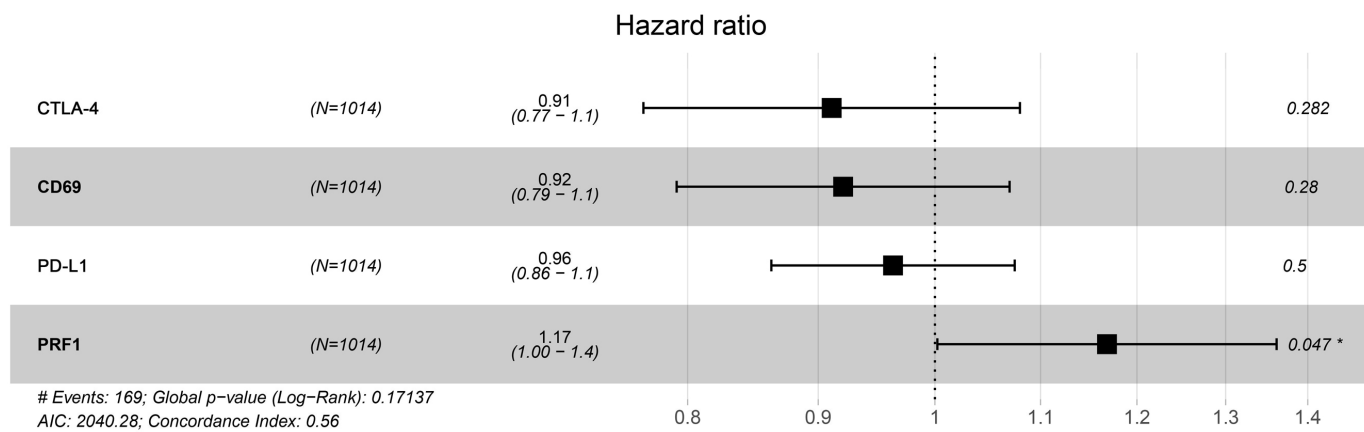

**Figure S6. Forest plotting of the hazard ratios of four potential hub genes. \*P<0.05.**

Supplement: Supplementary file 8 — Additional file 8. [file 12885_2021_9044_MOESM8_ESM.pdf]
