# Supplementary material for: Multi-omics analysis of an immune-based prognostic predictor in non-small cell lung cancer
Source: BMC Cancer. 2021 Dec 10;21:1322. doi: 10.1186/s12885-021-09044-4 (PMC8662860; doi:10.1186/s12885-021-09044-4)
Supplement: Supplementary file 5 — Additional file 5. [file 12885_2021_9044_MOESM5_ESM.pdf]

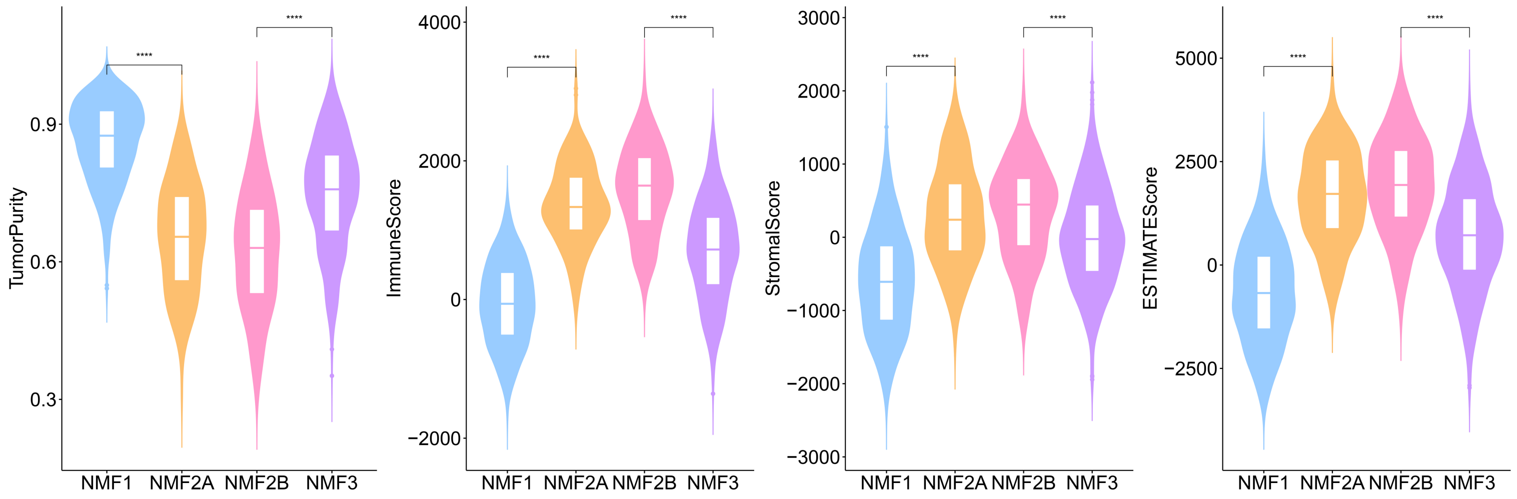

Figure S3. Diverse immune, stroma, and tumor purity scores of four immune subgroups. Comparisons were made between NMF1 and NMF2A, and between NMF2B and NMF3. ****P<0.0001.
