# Supplementary material for: Multi-omics analysis of an immune-based prognostic predictor in non-small cell lung cancer
Source: BMC Cancer. 2021 Dec 10;21:1322. doi: 10.1186/s12885-021-09044-4 (PMC8662860; doi:10.1186/s12885-021-09044-4)
Supplement: Supplementary file 6 — Additional file 6. [file 12885_2021_9044_MOESM6_ESM.pdf]

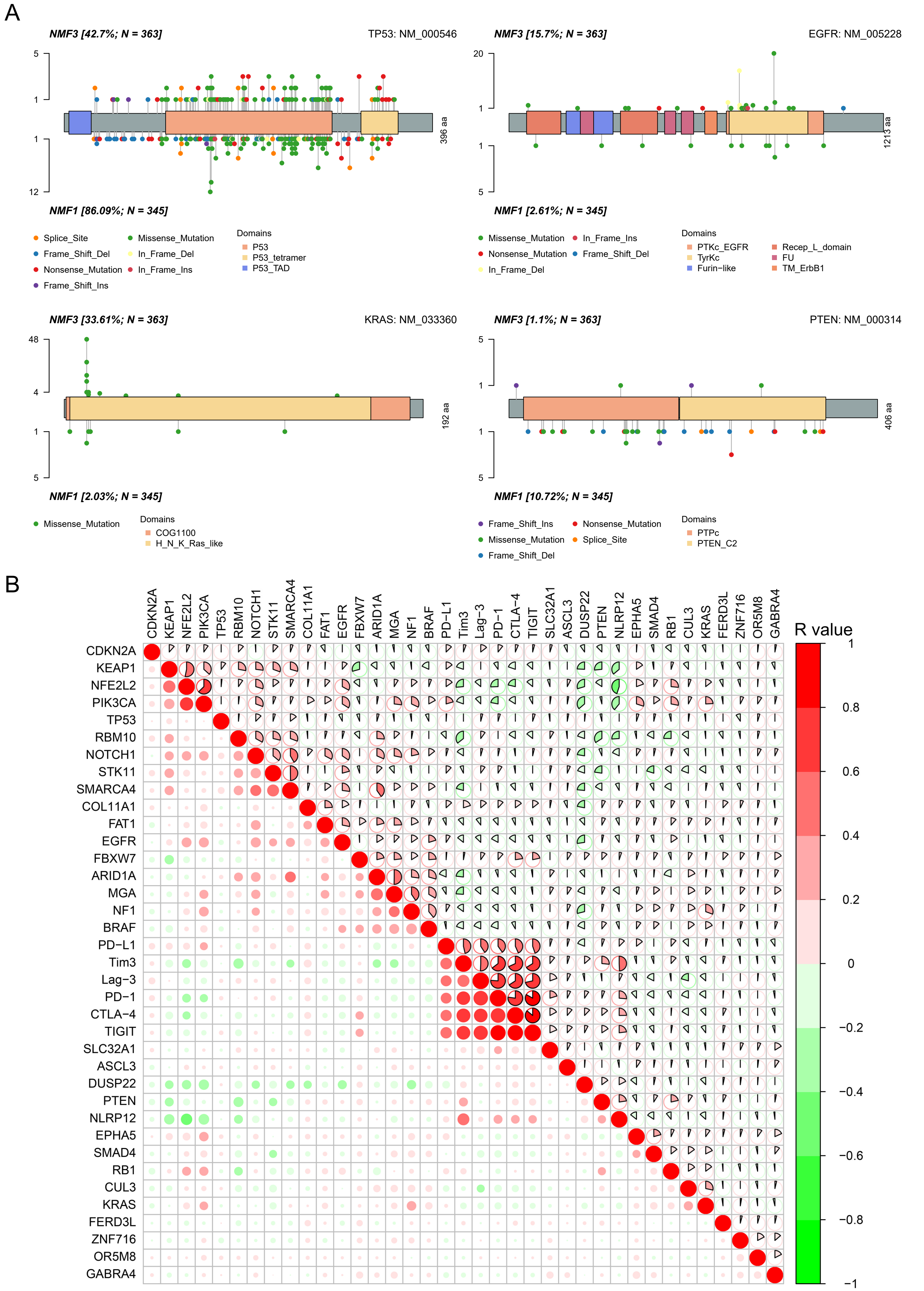

Figure S4. Additional mutational analyses in NMF subgroups.

(A) Detailed point mutations in TP53 and KRAS among different NMF subgroups; (B) Co-occurrence analysis revealed that most driver genes were not closely related to the six immune checkpoints.
