# Supplementary material for: Multi-omics analysis of an immune-based prognostic predictor in non-small cell lung cancer
Source: BMC Cancer. 2021 Dec 10;21:1322. doi: 10.1186/s12885-021-09044-4 (PMC8662860; doi:10.1186/s12885-021-09044-4)
Supplement: Supplementary file 7 — Additional file 7. [file 12885_2021_9044_MOESM7_ESM.pdf]

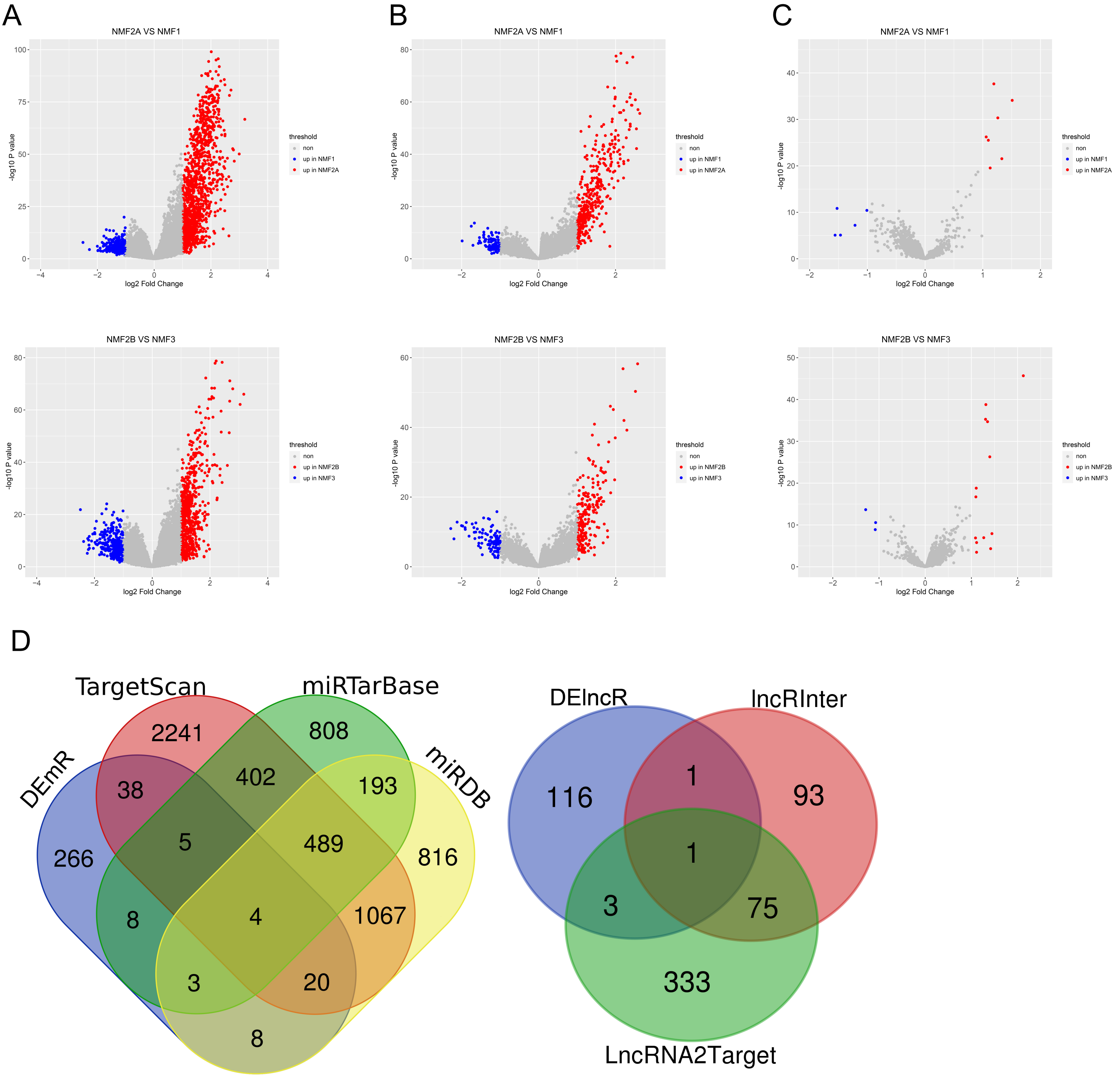

Figure S5. Differentially expressed RNAs (DERs) between NMF1 and NMF2A, or between NMF3 and NMF2B. Differentially expressed mRNAs (A), lncRNAs (B) and miRNAs (C) were identified between NMF2 and the other two groups with the criteria: |logFC| ＞1 and P <0.05; (D) Veen diagrams of differentially expressed mRNAs (DEmR)/lncRNAs (DElncR) with predicted miRNA-targeted mRNAs/lncRNAs using different databases, separately.
