# Supplementary material for: Multi-omics analysis of an immune-based prognostic predictor in non-small cell lung cancer
Source: BMC Cancer. 2021 Dec 10;21:1322. doi: 10.1186/s12885-021-09044-4 (PMC8662860; doi:10.1186/s12885-021-09044-4)
Supplement: Supplementary file 10 — Additional file 10. [file 12885_2021_9044_MOESM10_ESM.pdf]

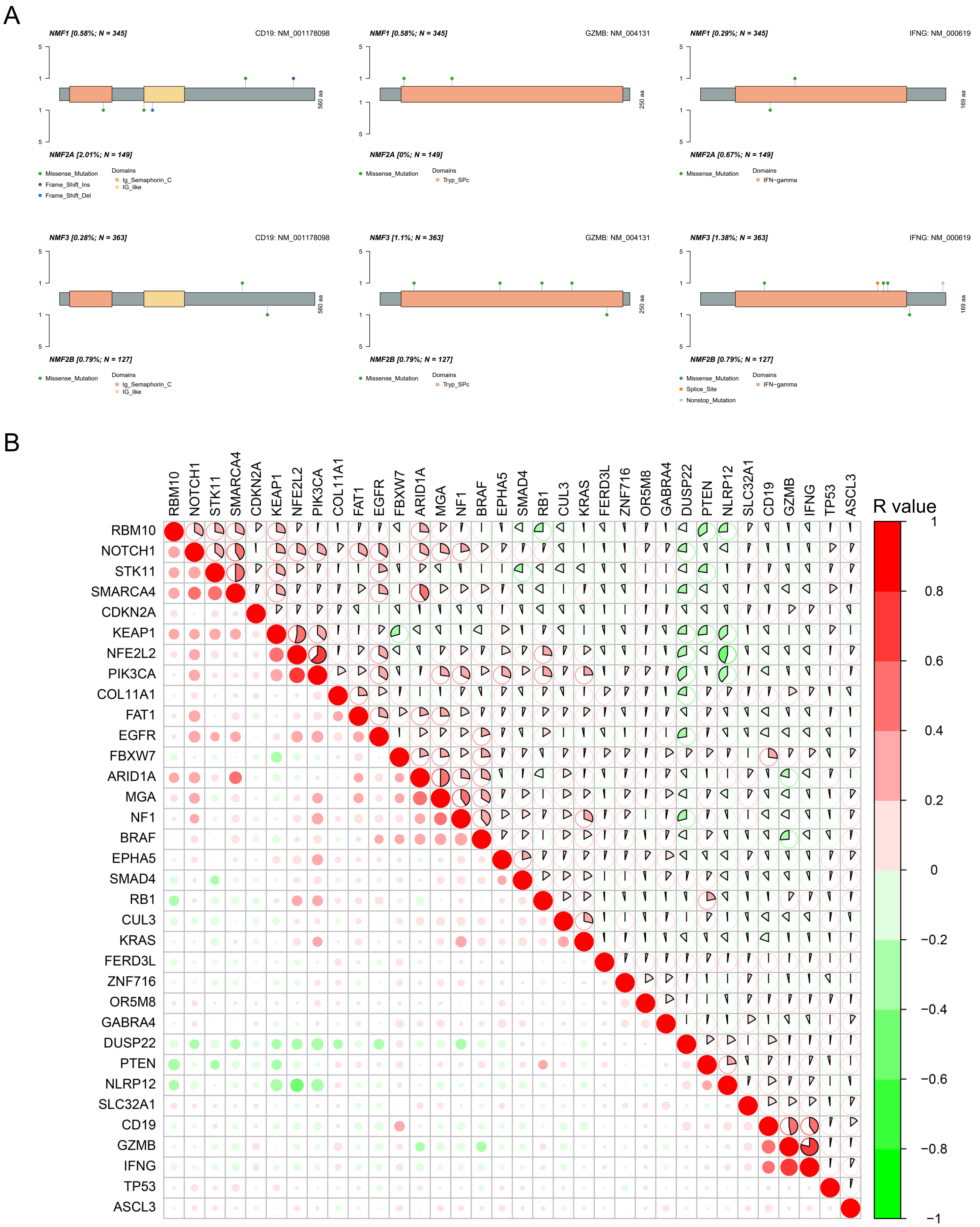

Figure S7. Additional mutation patterns of hub genes.
(A) Detailed point mutations of hub genes; (B) Few co-occurrences were identified between driver genes and hub genes.
