# Supplementary material for: Multi-omics analysis of an immune-based prognostic predictor in non-small cell lung cancer
Source: BMC Cancer. 2021 Dec 10;21:1322. doi: 10.1186/s12885-021-09044-4 (PMC8662860; doi:10.1186/s12885-021-09044-4)
Supplement: Supplementary file 11 — Additional file 11. [file 12885_2021_9044_MOESM11_ESM.pdf]

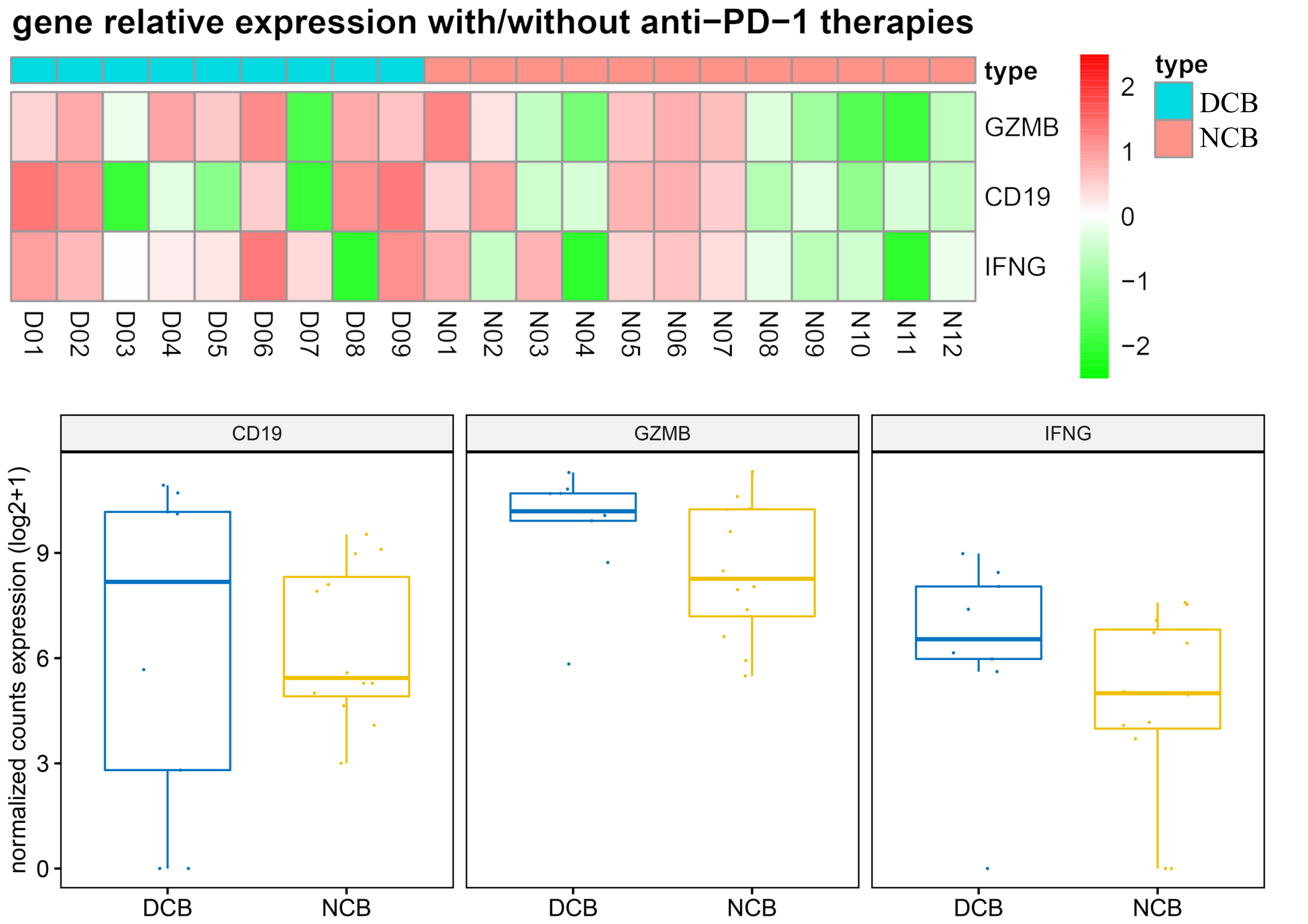

Figure S8. Expression of three hub genes between NSCLC patients with durable clinical benefit (DCB) and with non-durable benefit (NDB) after anti-PD-1 therapies in GSE136961.
