# Supplementary material for: Multi-omics analysis of an immune-based prognostic predictor in non-small cell lung cancer
Source: BMC Cancer. 2021 Dec 10;21:1322. doi: 10.1186/s12885-021-09044-4 (PMC8662860; doi:10.1186/s12885-021-09044-4)
Supplement: Supplementary file 1 — Additional file 1. [file 12885_2021_9044_MOESM1_ESM.pdf]

A

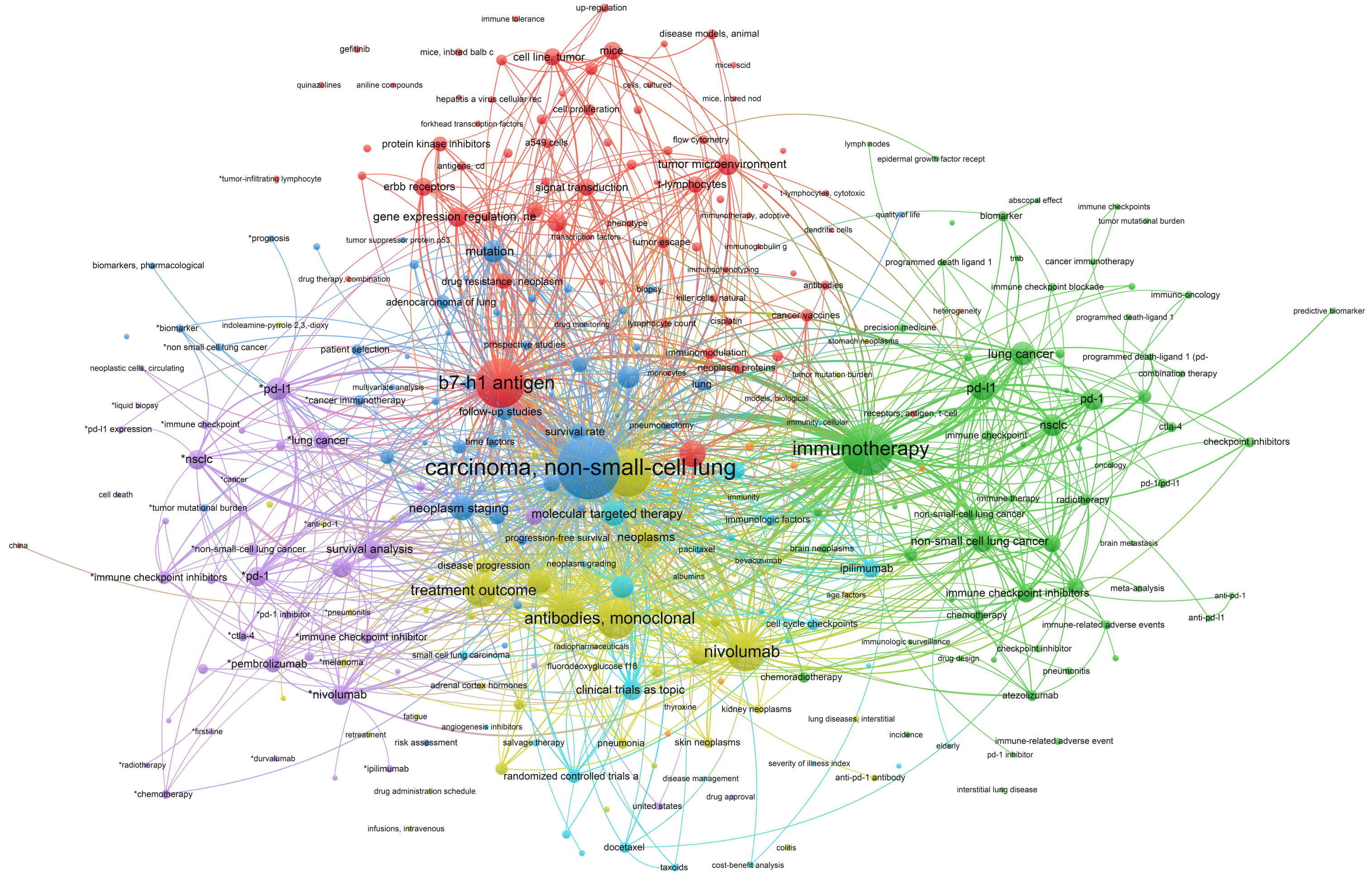

B

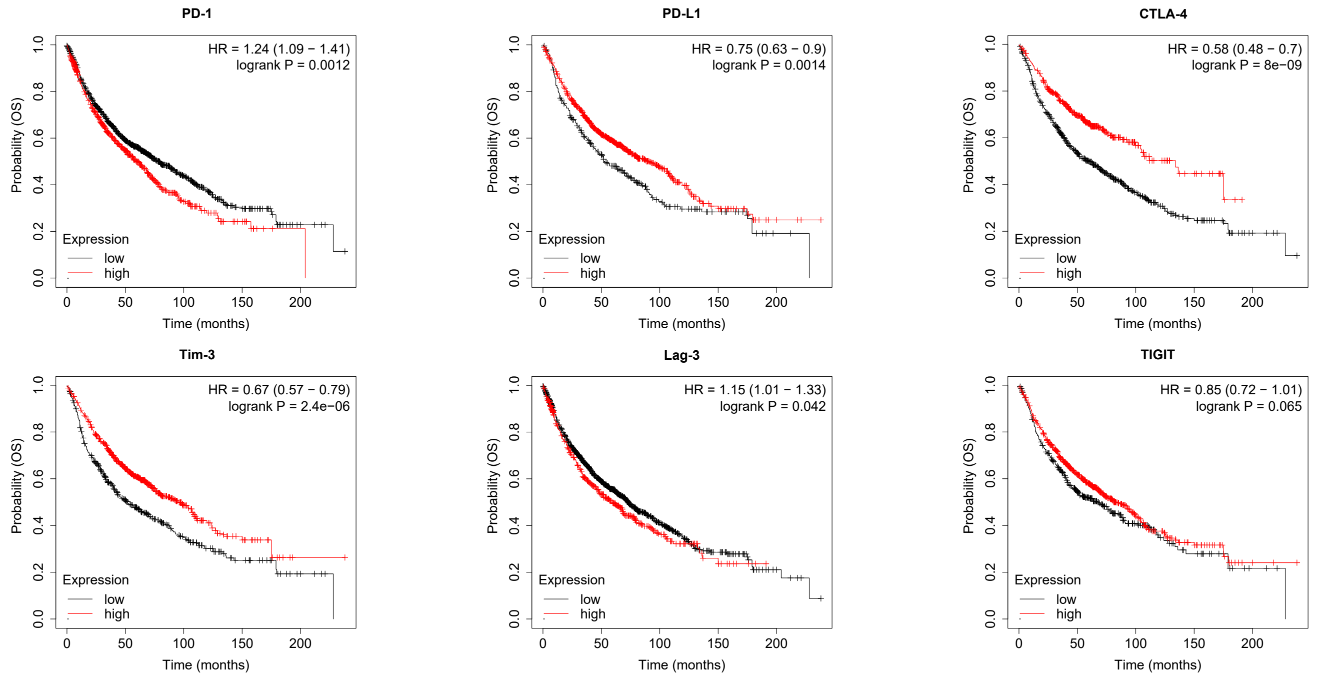

C

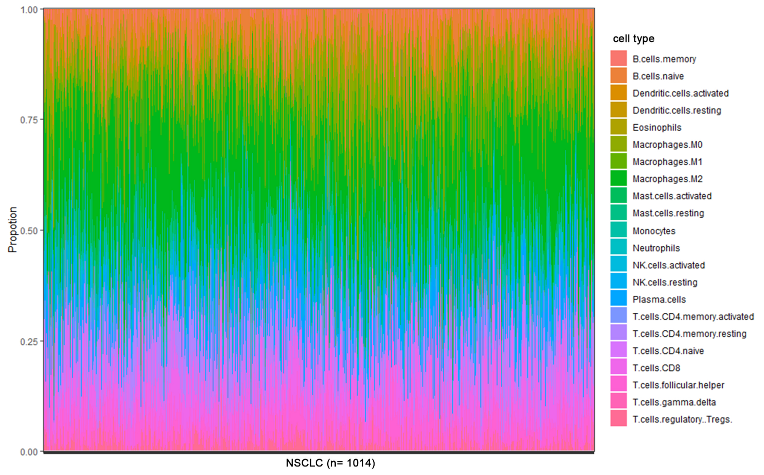

Figure S1. Background information concerning immune checkpoint inhibition in NSCLC.

(A) Keyword network in the field of NSCLC immunotherapy indicated research focus concerning immune checkpoint inhibition, especially PD-1/PD-L1 inhibition; (B) Inconsistent prediction power of overall survival status using the expression of each single immune checkpoint in the Kaplan Meier Plotter database, n=1144; (C) The intra-tumoral heterogeneity of the immune infiltration status indicated by the expression scores of 22 immune cell types in NSCLC patients using CIBERSORT, n=1014.
